# Correction: *Seriphidium herba-alba* (Asso): A comprehensive study of essential oils, extracts, and their antimicrobial properties

**DOI:** 10.1371/journal.pone.0336500

**Published:** 2025-11-07

**Authors:** Hazem Aqel, Husni Farah

There is an error in affiliation 1 for author Hazem Aqel. The correct affiliation 1 is: Basic Medical Sciences Department, College of Medicine, Al-Balqa Applied University, Salt, Jordan.
