# Supplementary material for: Seriphidium herba-alba (Asso): A comprehensive study of essential oils, extracts, and their antimicrobial properties
Source: PLoS One. 2024 Apr 25;19(4):e0302329. doi: 10.1371/journal.pone.0302329 (PMC11045107; doi:10.1371/journal.pone.0302329)

**S1 Fig.** *Seriphidium herba-alba (Asso)* composite, the Botanical Garden of Hashemite University, Zarqa, Jordan.


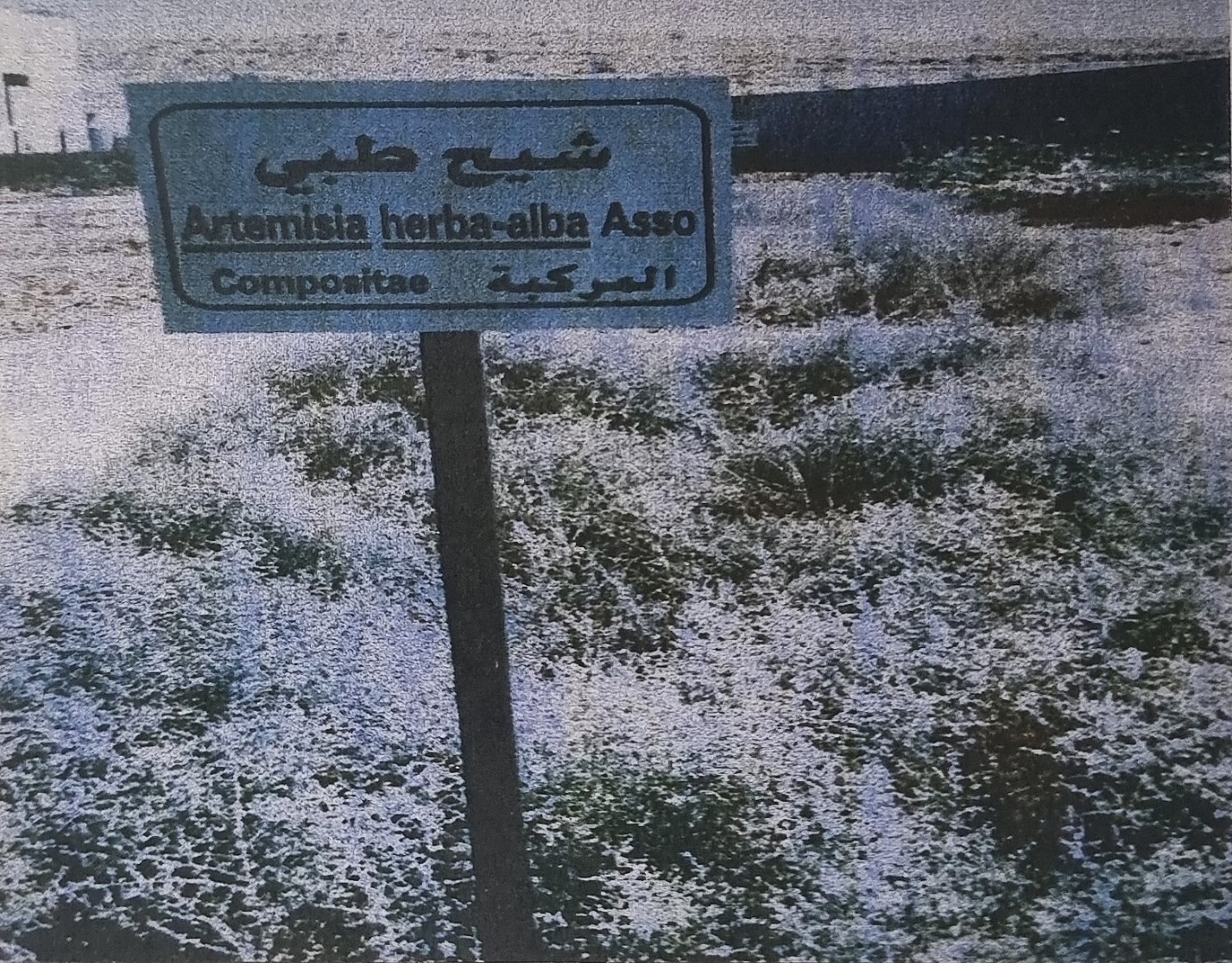

Supplement: S1 Fig — (DOCX) [file pone.0302329.s001.docx]
