# Supplementary material for: Seriphidium herba-alba (Asso): A comprehensive study of essential oils, extracts, and their antimicrobial properties
Source: PLoS One. 2024 Apr 25;19(4):e0302329. doi: 10.1371/journal.pone.0302329 (PMC11045107; doi:10.1371/journal.pone.0302329)
Supplement: S3 Fig — (DOCX) [file pone.0302329.s003.docx]

**S3 Fig.** Extraction of saponin fraction.
