# Supplementary material for: Seriphidium herba-alba (Asso): A comprehensive study of essential oils, extracts, and their antimicrobial properties
Source: PLoS One. 2024 Apr 25;19(4):e0302329. doi: 10.1371/journal.pone.0302329 (PMC11045107; doi:10.1371/journal.pone.0302329)
Supplement: S1 Table — (DOCX) [file pone.0302329.s005.docx]

**S1 Table.** Uses of *Seriphidium herba-alba (Asso)* composite.

| 1. Protection against body weight loss of diabetic animals. 2. Prevent elevation of glycosylated hemoglobin level. 3. Healing wounds and burns and stopping excessive bleeding. 4. Used by Bedouins as a healing plant by inhaling the smoke. 5. Used as cultivated ornaments. 6. Medicine like hot or cold drinks (extremely bitter taste). 7. Perfumery and culinary herbs. 8. Phenolics produced by *Seriphidium herba-alba (Asso)* composite as a defense plant against microorganisms, insects, and herbivores. |
| --- |
