# Supplementary material for: Seriphidium herba-alba (Asso): A comprehensive study of essential oils, extracts, and their antimicrobial properties
Source: PLoS One. 2024 Apr 25;19(4):e0302329. doi: 10.1371/journal.pone.0302329 (PMC11045107; doi:10.1371/journal.pone.0302329)
Supplement: Attachment — Submitted filename: PONE-D-23-41380_reviewer (Recovered).pdf [file pone.0302329.s006.pdf]

PLOS ONE

Artemisia herba alba: A Comprehensive Study of Essential Oils, Extracts, and Their Antimicrobial Properties

--Manuscript Draft--

**Manuscript Number:**	PONE-D-23-41380
**Article Type:**	Research Article
**Full Title:**	Artemisia herba alba: A Comprehensive Study of Essential Oils, Extracts, and Their Antimicrobial Properties
**Short Title:**	Antibacterial and antifungal activity of Artemisia herba alba extracts
**Corresponding Author:**	Hazem Aqel, Ph.D.<br>Al-Balqa' Applied University<br>Salt, JORDAN
**Keywords:**	Artemisia herba alba, essential oil, antimicrobial activity, phytochemical analysis, natural extracts.
**Abstract:**	Artemisia herba alba, a plant celebrated for its therapeutic qualities, is widely used in traditional medicinal practices throughout the Middle East and North Africa. The study conducted a detailed analysis of the chemical composition of essential oils and various extracts. This research also aimed to assess the antimicrobial capabilities of these components and to establish a connection between the chemical makeup of the extracts and their biological effectiveness. Essential oils were characterized by mass spectrometry and retention index methods. Several extraction methods were used to obtain phenolic and saponin compounds from different plant parts, and their yields were compared. Antibacterial and antifungal activities were evaluated using agar diffusion and MIC tests against various pathogens. Analysis of essential oils revealed a complex profile with α-thujone, β-thujone, and santolina alcohol as the main components. Significant variability in extraction yields was observed depending on the method and plant part. The extracts showed selective antibacterial activity, particularly against Staphylococcus aureus, and broad-spectrum antifungal activity, particularly against Candida albicans and Aspergillus spp. - Soaked methanol extract showed the strongest antimicrobial properties. Artemisia herba alba extracts possess remarkable antimicrobial properties influenced by their chemical composition. The results of this study demonstrate the potential of Artemisia herba alba as a natural antimicrobial source with implications for pharmaceutical applications. Future research should focus on isolating specific bioactive compounds and conducting further in vivo testing.
**Order of Authors:**	Hazem Aqel, Ph.D.
	Husni Farah, Ph.D
**Additional Information:**	
**Question**	**Response**
**Financial Disclosure**<br><br>Enter a financial disclosure statement that describes the sources of funding for the work included in this submission. Review the submission guidelines for detailed requirements. View published research articles from *PLOS ONE* for specific examples.<br><br>This statement is required for submission and **will appear in the published article** if the submission is accepted. Please make	The author(s) received no specific funding for this work.

sure it is accurate.<br><br>**Funded studies**<br>Enter a statement with the following details:<br>• Initials of the authors who received each award<br>• Grant numbers awarded to each author<br>• The full name of each funder<br>• URL of each funder website<br>• Did the sponsors or funders play any role in the study design, data collection and analysis, decision to publish, or preparation of the manuscript?<br><br>Did you receive funding for this work?	
**Competing Interests**<br><br>Use the instructions below to enter a competing interest statement for this submission. On behalf of all authors, disclose any competing interests that could be perceived to bias this work—acknowledging all financial support and any other relevant financial or non-financial competing interests.<br><br>This statement is required for submission and **will appear in the published article** if the submission is accepted. Please make sure it is accurate and that any funding sources listed in your Funding Information later in the submission form are also declared in your Financial Disclosure statement.<br><br>View published research articles from *PLOS ONE* for specific examples.	The authors have declared that no competing interests exist.

**NO authors have competing interests**<br><br>Enter: *The authors have declared that no competing interests exist.*<br><br>**Authors with competing interests**<br><br>Enter competing interest details beginning with this statement:<br><br>*I have read the journal's policy and the authors of this manuscript have the following competing interests: [insert competing interests here]*<br><br>* typeset	
**Ethics Statement**<br><br>Enter an ethics statement for this submission. This statement is required if the study involved:<br><br>• Human participants<br>• Human specimens or tissue<br>• Vertebrate animals or cephalopods<br>• Vertebrate embryos or tissues<br>• Field research<br><br>Write "N/A" if the submission does not require an ethics statement.<br><br>General guidance is provided below. Consult the submission guidelines for detailed instructions. **Make sure that all information entered here is included in the Methods section of the manuscript.**	Not applicable.

**Format for specific study types**<br><br>**Human Subject Research (involving human participants and/or tissue)**<br>• Give the name of the institutional review board or ethics committee that approved the study<br>• Include the approval number and/or a statement indicating approval of this research<br>• Indicate the form of consent obtained (written/oral) or the reason that consent was not obtained (e.g. the data were analyzed anonymously)<br><br>**Animal Research (involving vertebrate animals, embryos or tissues)**<br>• Provide the name of the Institutional Animal Care and Use Committee (IACUC) or other relevant ethics board that reviewed the study protocol, and indicate whether they approved this research or granted a formal waiver of ethical approval<br>• Include an approval number if one was obtained<br>• If the study involved *non-human primates*, add *additional details* about animal welfare and steps taken to ameliorate suffering<br>• If anesthesia, euthanasia, or any kind of animal sacrifice is part of the study, include briefly which substances and/or methods were applied<br><br>**Field Research**<br><br>Include the following details if this study involves the collection of plant, animal, or other materials from a natural setting:<br>• Field permit number<br>• Name of the institution or relevant body that granted permission	
**Data Availability**<br><br>Authors are required to make all data underlying the findings described fully available, without restriction, and from the time of publication. PLOS allows rare exceptions to address legal and ethical concerns. See the PLOS Data Policy and FAQ for detailed information.	Yes - all data are fully available without restriction

Powered by Editorial Manager® and ProduXion Manager® from Aries Systems Corporation

A Data Availability Statement describing where the data can be found is required at submission. Your answers to this question constitute the Data Availability Statement and **will be published in the article**, if accepted.<br><br>**Important:** Stating 'data available on request from the author' is not sufficient. If your data are only available upon request, select 'No' for the first question and explain your exceptional situation in the text box.<br><br>Do the authors confirm that all data underlying the findings described in their manuscript are fully available without restriction?	
**Describe where the data may be found in full sentences. If you are copying our sample text, replace any instances of XXX with the appropriate details.**<br><br>• If the data are **held or will be held in a public repository**, include URLs, accession numbers or DOIs. If this information will only be available after acceptance, indicate this by ticking the box below. For example: *All XXX files are available from the XXX database (accession number(s) XXX, XXX.).*<br>• If the data are all contained **within the manuscript and/or Supporting Information files**, enter the following: *All relevant data are within the manuscript and its Supporting Information files.*<br>• If neither of these applies but you are able to provide **details of access elsewhere**, with or without limitations, please do so. For example:<br><br>*Data cannot be shared publicly because of [XXX]. Data are available from the XXX Institutional Data Access / Ethics Committee (contact via XXX) for researchers who meet the criteria for access to confidential data.*<br><br>*The data underlying the results presented in the study are available from (include the name of the third party*	All relevant data are within the manuscript and its Supporting Information files.

and contact information or URL).<br>• This text is appropriate if the data are owned by a third party and authors do not have permission to share the data.<br><br>* typeset	
Additional data availability information:	Tick here if the URLs/accession numbers/DOIs will be available only after acceptance of the manuscript for publication so that we can ensure their inclusion before publication.

Powered by Editorial Manager® and ProduXion Manager® from Aries Systems Corporation

Manuscript Click here to access/download;Manuscript;Manuscript.docx

Artemisia herba alba: A Comprehensive Study of Essential Oils, Extracts, and Their Antimicrobial Properties

Hazem Aqel[1]*and Husni Farah[2]¶,

[1*] Basic Medical Sciences Department, Al-Balqa' Applied University, Salt, Jordan
[2] Medical Laboratory Sciences Department, Al-Ahliyya Amman University, Amman, Jordan
[3] Chemistry Department, Jordan University, Amman, Jordan

*Corresponding author
E-mail: hazem.aqel@bau.edu.jo (HA)

Contributing authors: h.farah@amman.edu.jo

¶This author contributed equally to this work.

Abstract

Artemisia herba alba, a plant celebrated for its therapeutic qualities, is widely used in traditional medicinal practices throughout the Middle East and North Africa. The study conducted a detailed analysis of the chemical composition of essential oils and various extracts. This research also aimed to assess the antimicrobial capabilities of these components and to establish a connection between the chemical makeup of the extracts and their biological effectiveness. Essential oils were characterized by mass spectrometry and retention index methods. Several extraction methods were used to obtain phenolic and saponin compounds from different plant parts, and their yields were compared. Antibacterial and antifungal activities were evaluated using agar diffusion and MIC tests against various pathogens. Analysis of essential oils revealed a complex profile with α-thujone, β-thujone, and santolina alcohol as the main components. Significant variability in extraction yields was observed depending on the method and plant part. The extracts showed selective antibacterial activity, particularly against *Staphylococcus aureus*, and broad-spectrum antifungal activity, particularly [illegible]*nst Candida albicans* and *Aspergillus spp*. - Soaked methanol extract showed the strongest antimicrobial properties. *Artemisia herba alba* extracts possess remarkable antimicrobial properties influenced by their chemical composition. The results of this study demonstrate the potential of *Artemisia herba alba* as a natural antimicrobial source with implications for pharmaceutical applications. Future research should focus on isolating specific bioactive compounds and conducting further *in vivo* testing.

Keywords: *Artemisia herba alba*, essential oil, antimicrobial activity, phytochemical analysis, natural extracts.

Introduction

White wormwood, known scientifically as *Artemisia herba alba*, is a perennial shrub indigenous to North Africa and the Middle East. It is widely recognized for its use in traditional medicine. Numerous studies have explored its diverse pharmacological properties, particularly focusing on its antibacterial and antifungal effects [1]. The essential oil extracted from this plant is rich in a variety of volatile compounds, which are believed to be the key contributors to its medicinal effectiveness [2].

Among the critical bioactive components found in *Artemisia herba alba* are phenolic and saponin compounds, typically derived from its aerial parts. These components demonstrate notable antimicrobial activities. However, their yield and composition are subject to variation and are influenced by factors such as the specific plant parts used, and the extraction methods applied [2,3]. Notably, the crude phenolic extracts from this species have shown significant effects against bacteria and fungi [4], which can be attributed to their antioxidant properties and their ability to disrupt the cell membranes of microbes [5,6].

In response to the growing problem of antibiotic-resistant bacteria, research efforts have intensified to discover new antimicrobial agents from plants [7]. *Artemisia herba alba* extracts have been found to be effective

against both gram-positive and gram-negative bacteria in various studies, highlighting their potential in combatting resistant bacterial strains, including *Staphylococcus aureus* and *Pseudomonas aeruginosa* [8].

The antifungal capabilities of *Artemisia herba alba* are equally significant, particularly considering the limitations and side effects of many synthetic antifungal drugs. Research has shown that *Artemisia* extracts can inhibit fungi such as *Candida albicans*, which cause infections in immunocompromised individuals [9], and their efficacy against molds such as *Penicillium* and *Aspergillus* suggests their potential use as natural food preservatives [7].

However, despite extensive studies, there is still much to learn about the comprehensive antimicrobial properties of *Artemisia herba alba*. Many research efforts have concentrated on isolated compounds or specific extraction techniques, often without comparing different extracts and their bioactivities [10,11]. In addition, there is an ongoing need to link the chemical makeup of these extracts to their antimicrobial effectiveness for a clearer understanding of their action mechanisms.

The primary goals of this study include: analyzing the chemical composition of *Artemisia herba alba*'s essential oils and extracts and identifying their major and minor components; comparing extraction yields from various plant parts using different methods; assessing the antibacterial efficacy of these extracts against a range of gram-positive and gram-negative bacteria; evaluating the antifungal effectiveness against common yeasts and molds; and examining the relationship between the chemical profiles of the extracts and their antimicrobial activity. This study deepen our understanding of *Artemisia herba alba*'s pharmacological potential, emphasizing its traditional medicinal role and its possible modern therapeutic applications.

Materials and Methods

Plant collection

Artemisia herba alba specimens were collected from the Botanical Garden of Hashemite University, Zarqa, Jordan. Prof. A. El-Oqlah performed taxonomic verification at the Biological Sciences Department, Yarmouk University, Irbid, Jordan. A representative sample was archived at the Medicinal Chemistry and Pharmacognosy Department, Jordan University of Science and Technology.

Microbial strains

In this study, the bacterial strains tested encompassed both gram-positive and gram-negative strains. Specifically, *Staphylococcus aureus* represented gram-positive bacteria, whereas the gram-negative group included *Escherichia coli*, *Pseudomonas aeruginosa*, *Klebsiella pneumoniae*, and *Proteus mirabilis*. In addition, the pathogenic yeast *Candida albicans* and various pathogenic molds such as *Penicillium*, *Fusarium*, *Aspergillus*,

and *Rhizopus* species were examined. These microbial strains were obtained from the microbiology laboratory at Al-Bashir Hospital in Amman, Jordan.

Preparation of plant material

The plant was air-dried to a constant weight in a shaded area and then pulverized through a 0.25 mm sieve using a Wiley mill. The resulting powder was stored in airtight glass containers at 5°C until extraction.

Extraction Methods

Soxhlet extraction

Aerial parts and flowers (20 g) were subjected to Soxhlet extraction using petroleum ether for phenolic compounds, methanol for saponins, n-hexane for lipids and terpenoids, ethyl acetate for more polar substances, and ethanol for polar compounds. Each solvent extract was concentrated at 40-50°C under vacuum, and the dry extracts were weighed and stored at -20°C.

Cold Extraction

Plant parts (50 g) were macerated in ethanol and methanol and separately stored for three weeks. Homogenization was concentrated at 40-50°C under reduced pressure. The residues were weighed and stored at -20°C.

Phenolic compound isolation

Twenty grams of powder of the aerial part was extracted with 200 mL petroleum ether for 12 h at 45°C, then filtered with cheesecloth, and the residue was collected. The residue was then extracted with 100 mL of absolute methanol for 6 h at 45°C. The methanolic extract was filtered and then concentrated to a small volume by evaporation under vacuum at 45°C. Fifty milliliters of ether were added to 50 mL of 10% sodium hydroxide and extracted using a separatory funnel. The aqueous layer was removed. Concentrated HCl was added to the remaining aqueous layer for acidification. Butanol (3X20 mL) was used to extract the aqueous layer using a separatory funnel. The same aqueous layer was extracted with petroleum ether (3x20 mL). Finally, the ether extract was dried using an evaporator. The butanol extract was added to the flask and evaporated using an evaporator [12]. The phenolic residue was weighed and stored in a freezer until further examination.

Detection was performed using a drop of 5% (w/v) $FeCl_2$ solution in distilled water added to the plant extract. Green was observed, confirming the detection of phenol. Detection can also be performed using an ethanolic KOH solution, which gives a yellow to phenolic compounds [12].

Saponin fraction isolation

Twenty grams of dried powder was shaken vigorously for 15 min in a 500-mL round-bottom flask (screwcap) with chloroform (3x70 mL) to exclude fatty substances. The extract was left separate, and the supernatant was completely decanted (filtration). Then, 80% methanol (200 mL) was added to the flask and boiled for 24 h using the Soxhlet method. The procedure involved filtering the extract into a 500 mL beaker using either cheesecloth or filter paper immediately after extraction. Following this, the filtrate was subjected to vacuum evaporation at 45°C. The remaining residue was then dissolved in 100 mL of distilled water. Subsequently, the water-soluble portion of the residue was extracted using butanol and applied in three separate 50 mL quantities. The sterilized filtrate was concentrated under vacuum at 40-45°C until a dry residue was obtained. The residue was weighed and stored in a freezer at -21°C until further examination [12].

The detection of saponin compounds was performed by adding a drop of chlorosulfonic acid to the extract. A reddish-brown color was observed, confirming saponin production [12].

Water extraction

Two methods were used to obtain water extraction: The first method was according to An et al. [13]. Water extraction was performed by pouring boiling distilled water (50 mL) into 5 g of dried powder. The mixture was left to stand for 15-20 min. The mixture was then filtered through a layer of cheesecloth, and the resulting filtrate was centrifuged at 6000 rpm for 15 min. The supernatant was removed and filtered through a Millipore microfilter (0.2 μm). The sterilized filtrate was concentrated under vacuum at 40-45°C until a dry residue was obtained. The residue was weighed and stored in a freezer at -21°C until further examination.

The second method was performed according to Al-Charchafchi et al. [14]. As mentioned previously, 50 mL of boiling distilled water was poured onto 5 g of dried powder. The process involved boiling the mixture for 10 min. After boiling, it was filtered through a layer of cheesecloth. The filtrate obtained from this step was then centrifuged at a speed of 6000 revolutions per minute for a duration of 15 min. After centrifugation, the supernatant was carefully separated and subsequently passed through a Millipore microfilter with a pore size of 0.2 μm for further purification. The sterilized filtrate was concentrated under vacuum at 40-45°C until a dry residue was obtained. The residue was weighed and stored in a freezer at -21°C until further examination.

GC/MS analysis

GC analyses were performed using an HP-5890 Series II instrument equipped with HPWAX and HP-5 capillary columns (30 m × 0.25 mm, 0.25 μm film thickness) and set to the following conditions: temperature program of 60°C for 10 min, followed by an increase of 5°C /min to 220 °C; injector and detector temperatures at 250 °C; carrier gas nitrogen (2 mL/min); detector dual FID; split ratio 1:30; and injection of standards of 0.5 μL). The chemicals were identified for both columns by comparing their retention times with those of pure, authentic samples and using their linear retention indices (LRI) relative to the series of n-hydrocarbons. The

relative proportions of each component, expressed as percentages, were determined by FID peak area normalization (mean of three replicates). GC/EIMS analyses were performed using a Varian CP-3800 gas chromatograph equipped with a DB-5 capillary column (30 m × 0.25 mm; coating thickness, 0.25 μm) and a Varian Saturn 2000 ion trap mass detector. Analysis conditions included: injector and transfer line temperatures at 220 and 240°C respectively; the oven temperature programed from 60°C to 240°C at 3°C /min; carrier gas helium at 1 mL/min; injection of 0.2 μL (10% hexane solution); split ratio 1:30. The identification of the components was based on comparing the retention times with those of authentic samples, comparing their linear retention indices relative to the series of n-hydrocarbons, and by computer matching against commercial (NIST 98 and ADAMS 95) and home-made library mass spectra built from pure substances and components of known essential oils [15,16].

Microbial inoculum and growth conditions

The bacterial inoculum was prepared from a nutrient broth culture incubated for 24 h at 37°C. The suspension density was adjusted to approximately 10^4 colony-forming units per mL by comparing its turbidity with that of a McFarland 0.5 $BaSO_4$ standard. The standard was prepared by adding 0.5 mL of 0.048 M $BaCl_2$ to 99.5 mL of 0.36 N H_2SO_4. Aliquots of 4 to 6 mL were dispensed into screwcap tubes and stored in the dark at room temperature. The bacterial suspension and McFarland tube were held side by side to adjust the turbidity and viewed against a backed background. The suspension was supplemented or diluted as required.

The antimicrobial activity of various plant extracts such as n-hexane, ethyl acetate, and ethanol extracts, as well as phenols, saponin, ethanol-soaked, methanol-soaked, and water extracts extracted from various parts of *Artemisia herba alba*, were analyzed separately using the disk agar diffusion method [55] and evaluated against the microorganisms mentioned above.

Disk agar diffusion method

The disks were impregnated with the various plant extracts to be tested, allowed to dry, and then placed on the plates within an hour of pouring. Before disk placement, the plate surface was inoculated with a swab dipped in the standardized bacterial suspension. The surface of the plates was wiped in three directions to ensure an even and complete distribution of the inoculum throughout the plate. Discs were placed on plates and incubated at 37°C for 24 h [17,18].

The diameter of the inhibition zones was measured in mm. For this experiment, each test was conducted in triplicate to ensure reliability and accuracy. The average of these three replicates was then calculated to obtain a more accurate and representative result. Standard antibiotics such as vancomycin 30 μL for S. aureus, streptomycin 10 μL for *E. coli*, *K. pneumoniae*, and *P. mirabilis*, carbenicillin 100 μL for *P. aerug[illegible]sa*, and amphotericin B 9.6 μg for fungi (yeast and molds) were used as ap[illegible]tive controls, and phosphate-buffered saline was used as a negative control.

Minimum inhibitory concentration (MIC) determination

In this study, different crude extracts of *Artemisia herba alba* were used to determine the minimum inhibitory concentration (MIC) against various microorganisms. To prepare each extract for testing, 100 mg of the extract was first dissolved in 1 mL of dimethyl sulfoxide (DMSO, supplied by Sigma). A series of twofold dilutions was then created. This was accomplished by adding 1 mL of the dissolved extract to test tubes containing an equal volume (1 mL) of phosphate-buffered saline (PBS) with a pH of 6.8. Bacterial cell suspensions were prepared and adjusted to give a final inoculum concentration of approximately 1×10^6 cells per mL (Spectronic Instruments, USA). The extract buffer tubes were inoculated with 0.5 mL of the prepared inoculum and incubated at 37^oC. The tubes were read after 24 h, and 0.1 mL of each test tube was spread on the surface of a nutrient agar plate and incubated at 37^oC for 24 h to count CFU/mL.

Results

Chemical profile of *Artemisia herba alba* essential oil: main components and retention indices

Table 1 provides a detailed chemical profile of the essential oil of *Artemisia herba alba* and a complex series of 52 identified compounds. These were predominantly identified using mass spectrometry and retention index methods, with some verified using pure reference compounds. The oil was characterized by several main components, including santolina alcohol, α-thujone, β-thujone, chrysanthenone, cis-chrysanthenyl acetate, 1,8-cineole, camphor, and limonene, which were present in significant amounts ranging from 6.34% to 18.12%. These key components were instrumental in defining the aromatic and potential therapeutic profiles of the oil. The listed compounds exhibited various polarities and volatilities, as indicated by their linear retention indices on the apolar and polar columns. While most components were in lower concentrations, some compounds were reported without reported percentages, suggesting trace amounts or unquantified presence. Overall, the essence of Artemisia herba alba is a rich complex of terpenes, alcohols, and ketones, reflecting the typical heterogeneity of essential oils used in various applications from the medical to the perfume industry.

Table 1. Chemical composition of *Artemisia herba alba* essential oil.

Components	LRI[a]	LRI[b]	Percentage	Identification
Santolina triene	910	1010	0.22	MS, RI[e]
α-thujene	932	1016	0.4	MS, RI, ST
α-pinene	941	1029	0.31	MS, RI, ST
Camphene	955	1071	1.99	MS, RI, ST
Sabinene	978	1112	0.43	MS, RI, ST
β-pinene	981	1126	0.33	MS, RI, ST
Yomogi alcohol	997	1401	0.18	MS, RI
α-terpinene	1020	1183	0.84	MS, RI, ST

p-cymene	1028	1274	0.81	MS, RI, ST
Limonene	1033	1198	6.34	MS, RI, ST
Santolina alcohol	1035	1413	13	MS, RI
β-phellandrene	1035	1199	0.23	MS, RI, ST
1,8-cineole	1039	1204	9.06	MS, RI, ST
γ-terpinene	1064	1252	0.84	MS, RI, ST
Cis-sabinene hydrate	1070	–	2.08	MS, RI, ST
Cis-linalool oxide	1075	–	Tr.[c]	MS, RI
Para-Mentha-2,4(8)-diene	1092	-	0.39	MS, RI, ST
Linalool	1101	1547	0.5	MS, RI, ST
Trans-sabinene hydrate	1103	–	0.86	MS, RI, ST
α-thujone	1106	1428	18.12	MS, RI, ST
β-thujone	1106	1446	10.06	MS, RI, ST
Trans-thujone	1118		2	MS, RI
Fenchol	1123	1584	3.86	MS, RI
Chrysanthenone	1125	–	11.57	MS, RI
Cis-p-menth-2-en-1-ol	1127	–	1.33	MS, RI
Cis-p-menth-2,8-dien-1-ol	1135	–	0.41	MS, RI
Camphor	1145	1522	8.75	MS, RI, ST
Cis-sabinol	1153	–	2.32	MS, RI
Cis-chrysanthenol	1164	–	7.96	MS, RI
Borneol	1175	1796	1.47	MS, RI, ST
Umbellulone	1176	–	0.45	MS, RI, ST
4-terpinenol	1182	1607	2.33	MS, RI, ST
p-cymen-8-ol	1185	1838	Tr.	MS, RI
Neo-verbanol	1186	–	1.3	MS, RI, ST
α-terpinenol	1193	1698	2.54	MS, RI, ST
γ-terpinenol	1208	–	0.94	MS, RI, ST
Verbenone	1208	1716	2.09	MS, RI, ST
Carvone	1246	1741	1.8	MS, RI, ST
Carvenone	1251	–	1.58	MS, RI, ST
Cis-chrysanthenyl acetate	1254	–	11.94	MS, RI, ST
Trans-Ascaridol glycol	1259	–	0.78	MS, RI, ST
Trans-carvone oxide	1267	–	0.36	MS, RI, ST
Perilla aldehyde	1272	–	1.04	MS, RI, ST
Isobornyl acetate	1285	1582	3.15	MS, RI
Thymol	1292	2187	0.34	MS, RI, ST
Carvacrol	1298	2219	0.46	MS, RI, ST
β-caryophyllene	1419	1604	2.52	MS, RI, ST
Germacrene D	1491	1691	4.12	MS, RI
Bicyclogermacrene	1496	1493	2.64	MS, RI
δ-cadinene	1524	1731	0.3	MS, RI, ST
Caryophyllene oxide	1578	2071	1.76	MS, RI, ST

β-eudesmol	1651	–	4.2	MS, RI
Total identified			52	

[a] Linear retention index (apolar column)

[b] Linear retention index (polar column)

[c] trace amounts < 0.1

[e] identification: MS=mass spectrometry, RI=retention index, ST=pure reference compound

Extraction yields from *Artemisia herba alba*: comparative analysis of phenolic and saponin compounds

Table 2 shows the extraction yields from different parts of the *Artemisia herba alba* plant, with all initial samples weighing 20 g. The extraction methods examined resulted in different extract weights, which were categorized according to the type of extract and the part of the plant used.

For crude phenolic extracts, aerial parts with flowers gave a variable weight percentage between 4.40% and 13.80%, with an average of 9.02%. The median weight percentage for these extracts was 8.10%, with the most common value being 5.15%. In contrast, immature flowers alone gave a significantly higher yield of crude phenolic extract of 13.8%, whereas mature flowers gave a yield of 8.1%.

Crude saponin extracts were also derived from different plant parts. The combined aerial parts with flowers provided a mean extract weight of 5.89%, with a range of 4.95%–7.32% and a median of 5.66%. However, the aerial parts without flowers yielded 5.14%, immature flowers yielded 7.32%, and mature flowers yielded the lowest yield at 4.95%.

Other extraction methods were used on aerial parts with flowers, resulting in consistent yields across single measurements. The ethyl acetate and ethanol extracts had uniform weights of 9.15% and 7.67%, respectively. Soaking the aerial parts in methanol gave the highest extract weight at 17.75%, whereas ethanol soaking gave 15.53%. The two different water extraction methods each yielded 1%.

Notably, some extracts have detailed statistical data, such as crude saponin from aerial parts with flowers, indicating multiple measurements and variations in the extraction process. However, for several extracts, only single values were provided without additional statistical data, suggesting that either a single measurement was taken, or the variability needed to be documented.

The efficiency and yield of the extraction process depend on both the plant part being processed and the extraction method used. The table provides an overview of how different techniques and plant parts contribute to the yield of *Artemisia herba alba* extracts.

Table 2. Various extracts of *Artemisia herba alba*. The weight of the plant parts was 20 g.

Part of the plant tested	Type of extract	Weight of the	Mean (%)	Standard Deviation (%)	Min (%)	Median (%)	Max (%)

		extract (%)					
Aerial part with flowers	1. Crude phenolics	5.15	9.02	4.40	5.15	8.10	13.80
Immature flowers	2. Crude phenolics	13.8					
Mature flowers	3. Crude phenolics	8.1					
Aerial part with flowers	4. Crude saponin	6.17	5.89	1.09	4.95	5.66	7.32
Aerial part without flowers	5. Crude saponin	5.14					
Immature flowers	6. Crude saponin	7.32					
Mature flowers	7. Crude saponin	4.95					
Aerial part with flowers	8. Ethyl acetate	7.67	9.15	-	9.15	9.15	9.15
Aerial part with flowers	9. Ethanol	9.15	7.67	-	7.67	7.67	7.67
Aerial part with flowers	10. Soaked in methanol	17.75	15.53	-	15.53	15.53	15.53
Aerial part with flowers	11. Soaked in ethanol	15.53	17.75	-	17.75	17.75	17.75
Aerial part with flowers	12. Water extraction method 1	1	1.00	-	1.00	1.00	1.00
Aerial part with flowers	13. Water extraction method 2	1	1.00	-	1.00	1.00	1.00

Spectrum of antibacterial efficacy: *Artemisia herba alba* extracts against common pathogens

Table 3 evaluates the antibacterial efficacy of various extracts from *Artemisia herba alba* using agar diffusion and minimum inhibitory concentration (MIC) methods against a spectrum of bacteria. The findings reveal that the plant extracts exhibit a range of activities against both gram-positive and gram-negative bacteria, with varying levels of effectiveness.

Several extracts demonstrated antibacterial action against the gram-positive bacterium *S. aureus*, indicated by inhibition zones measuring between 11 and 20 mm. In particular, extracts 2, 4, 5, 6, 7, 8, and 9 showed inhibition, with the most potent being extract 10. The MIC values complemented these findings, with some extracts displaying substantial bacteriostatic properties at concentrations as low as 3.125 μg/mL. Notably, extracts 1, 3, 12, and 13 did not inhibit S. aureus, suggesting that they lack the necessary components or concentrations to affect this bacterium.

Regarding gram-negative bacteria, the extracts generally exhibited less activity. *E. coli* was resistant to most extracts, except for extract 10, which inhibited growth with an inhibition zone of 13 mm and a MIC of 3.125 μg/mL. *P. aeruginosa* was only susceptible to extract 1, which displayed a moderate inhibition zone and MIC indicative of moderate effectiveness. For *P. mirabilis*, extracts 10 and 11 were effective, with extract 10 showing a particularly significant inhibition zone and a corresponding MIC value. *K. pneumoniae* was similarly affected by extracts 10 and 11, with notable inhibition zones and MIC values, although the MIC for extract 10 against this bacterium was relatively high at 50 μg/mL.

The positive control, which likely represents a standard antibiotic, consistently inhibited all tested bacterial strains, affirming the validity of the assay. Conversely, the negative control exhibited no antibacterial activity, as expected for a control. Including standard error values with the inhibition zones suggests that the experiments were conducted multiple times to de[illegible]ine reliability, reflecting the variability often inherent in biological testing.

Overall, the results from Table 3 demonstrate that *Artemisia herba alba* extracts have selective antibacterial properties, with certain extracts, particularly extract 10, showing broad-spectrum effectiveness. The variation in activity across different extracts highlights the importance of extract type and specific bacterial strain when considering the potential therapeutic applications of these plant extracts.

Table 3. Antibacterial activity of various extracts of *Artemisia herba alba* by agar diffusion and MIC methods.

Type of Extract	Antibacterial activity									
	Gram-positive		Gram-negative							
	S. aureus		*E. coli*		*P. aeruginosa*		*P. mirabilis*		*K. pneumonia*	
	DDM*	MIC	DDM*	MIC	DDM*	MIC	DDM*	MIC	DDM*	MIC
1	0		0		0		16±0.4	12.5	0	
2	11±0.2	3.125	0		15±0.3	3.125	0		0	
3	0		0		0		0		0	
4	17±0.45	3.125	0		0		0		0	
5	18±0.3	3.125	0		0		0		0	
6	15.5±0.35	6.25	0		0		0		0	
7	15±0.3	3.125	0		0		0		0	
8	15±0.6	3.125	0		0		0		0	
9	16±0.07	50	0		11±0.2	3.125	0		0	
10	20±0.05	6.25	13±0.05	3.125	11±0.04	3.125	16±0.08	12.5	17±0.05	50
11	15±0.05	50	0		13±0.04	50	11±0.2	3.125	11±0.2	25
12	0		0		0		0		0	
13	0		0		0		0		0	
Positive control	12		13		13		13		13	
Negative control	0		0		0		0		0	

DDM, disc diffusion method; MIC, minimal inhibition concentration; *Inhibition zone in mm ± SE (S.E., Standard error).

Antifungal potency of *Artemisia herba alba* extracts in inhibition zones and minimum inhibitory concentrations

Table 4 provides a comprehensive overview of the antifungal activities of various extracts from *Artemisia herba alba*, as determined by the agar diffusion method and MIC tests against various fungi, including yeasts and molds.

The extracts showed varying degrees of efficacy against *C. albicans*, a common yeast, with extract 6 exhibiting the most potent activity, as evidenced by a 23-mm inhibition zone and an MIC value of 3.125 µg/ml. Other extracts, such as extract 2, demonstrated significant activity with a 20-mm zone and a higher MIC of 25 µg/mL. Extracts 4, 12, and 13 inhibited *C. albicans* growth.

In the context of molds, specifically *Penicillium spp*., only a subset of the extracts, notably extract 10, showed substantial inhibitory action with a 23 mm zone and a low MIC, indicating robust antifungal properties. Conversely, the majority of the extracts were ineffective against these mold species.

When tested against *Fusarium spp*., another mold type, seve[illegible]xtracts, particularly extract 9, were effective, displaying a 21 mm inhibition zone and a MIC of 3.125 µg/mL, suggesting a strong antifungal effect at a low concentration. Similar activity patterns were observed against *Aspergillus spp*., with extract 9 again showing the highest activity marked by an 18 mm zone and a low MIC.

The activity against Rhiz[illegible] spp., a mold known for its rapid growth, was notable in extracts 5, 8, 9, 10, and 11, with extracts 9 and 10 displaying the largest zones of inhibition at 24 mm. The MIC values for these extracts varied, indicating differences in their effectiveness in inhibiting fungal growth.

As benchmarks, the positive control's consistent inhibition across all fungi confirmed the assay's effectiveness. In contrast, the negative control confirmed the absence of inherent antifungal properties in the medium used for testing. The standard error values associated with the inhibition zones suggest that the measurements were repeated to ensure precision, which is typical in biological assays to account for variability.

Overall, the findings from Table 4 underscore the potential of *Artemisia herba alba* extracts as antifungal agents, with certain extracts, particularly numbers 9 and 10, demonstrating broad-spectrum antifungal properties against various fungal pathogens. The data indicate that the efficacy of the extracts can be species-specific and suggest a need for further research to isolate and understand the active compounds contributing to these antifungal effects.

Table 4. Antifungal activity of various extracts of *Artemisia herba alba* by agar diffusion and MIC

Type of extract	Antifungal activity									
	Yeast		Molds							
	Candida albicans		*Penicillium spp.*		*Fusarium spp.*		*Aspergillus spp.*		*Rhizopus spp.*	
	DDM*	MIC	DDM*	MIC	DDM*	MIC	DDM*	MIC	DDM*	MIC
1	13±0.48	6.125	0		16±0.01	6.25	0		5.43±0.3	3.125
2	20±0.05	25	0		20±0.001	6.25	0		4.99±0.23	3.125
3	11.3±0.04	3.125	0		15±0.3	6.25	0		7.69±0.14	3.125
4	5±0.3		0		0		0		0	
5	15±0.3	6.25	6.4±0.81	6.25	14±0.55	6.25	4.5±0.24	12.5	4.09±0.23	6.25
6	23±0.04	3.125	0	0	0	0	0		0	
7	14±0.05	6.25	0	0	0	0	0		0	
8	13.5±0.05	6.25	10±0.85	6.25	14±003	6.25	9.36±0.14	12.5	10±0.04	12.5
9	15±0.3	50	19.2±0.81	3.125	21±0.1	3.125	18±0.26	3.125	24±0.4	3.125
10	20±0.05	6.25	23±0.26	3.125	22±0.07	3.125	22±0.64	6.25	24±0.95	6.25
11	16±0.07	50	20±0.41	3.25	20±0.5	6.25	19±0.81	6.25	24±0.65	3.125
12	0		0		0		0		0	
13	0		0		0		0		0	
Positive Control	12		12		12		12		12	
Negative control	0		0		0		0		0	

DDM: Disc diffusion method; MIC: Minimal inhibition concentration; *Inhibition zone in mm ± SE (S.E., Standard error).

Discussion

The present study explores the field of ethnopharmacology and phytochemistry, focusing on *Artemisia herba alba*, a medicinal plant. This study aligns with the growing interest in natural products for their potential therapeutic applications, particularly as antimicrobial agents. It delves into the extraction, chemical analysis, and evaluation of antimicrobial properties of various extracts from *Artemisia herba alba*, a plant well-known in traditional medicine.

This study also contributes to new insights into the chemical composition and bioactivity of *Artemisia herba alba*, particularly regarding its essential oils and extracts. The study stands out in its comprehensive approach, examining a wide range of extracts and their efficacy against both bacterial and fungal pathogens. It introduces new data on the efficacy of different extraction methods, the specific bioactivity of various extracts, and their minimum inhibitory concentrations against a spectrum of microorganisms. This includes both common pathogenic bacteria and fungi, offering a broad perspective on the plant's antimicrobial potential.

The chemical composition of the essential oil (Table 1) reveals a rich tapestry of volatile compounds, with α-thujone, β-thujone, and santolina alcohol as the predominant constituents. These findings align with those of Ouguirti et al. [19] and Houti et al. [20], who reported similar profiles in their studies of *Artemisia* species, emphasizing the consistency in essential oil composition across different geographical locations.

The extraction yields (Table 2) varied significantly depending on the part of the plant and the extraction method, which is in accordance with the results obtained by Brendler et al. [21] and corroborates the findings of Adam et al. [22] who noted that the extraction method profoundly affects the yield and composition of plant extracts.

The antibacterial activities (Table 3) against both gram-positive and gram-negative bacteria were noteworthy, particularly for extracts 9 (ethanol) and 10 (soaked in methanol), which showed broad-spectrum activity. These results are consistent with those of Liu et al. [23] and Bisht et al. [24], who observed substantial activity of *Artemisia* extracts against *S. aureus* and *P. aeruginosa*. However, our results suggest higher efficacy than the minimal inhibition concentrations reported by Ahameethunisa et al. [25].

Antifungal activities (Table 4) against a range of fungi, including *C. albicans* and *Aspergillus spp*. Showcase some extracts with significant inhibitory properties. This is particularly interesting compared with the moderate activity reported by Mehani et al. [26], suggesting that the specific strains of *Artemisia herba alba* used in this study may possess unique or more potent antifungal compounds.

The potent antibacterial activity of crude phenolic extracts from immature flowers *against K. pneumonia*e aligns with previous studies that have documented the antibacterial properties of phenolic compounds [27,28]. The stronger antifungal efficacy against *C. albicans* is particularly noteworthy because it

adds to the evidence supporting the use of phenolics as antifungal agents [29,30]. The low MIC observed for mature flower extracts against *S. aureus* and *C. albicans* is consistent with the known bioactive profiles of mature plant parts, as reported in other studies [31-33].

Our results regarding crude saponin extracts are consistent with the literature that acknowledges their antimicrobial properties [34,35]. The findings that extracts from both the aerial parts with and without flowers possess significant antibacterial effects against *S. aureus* corroborate previous research [36,37], while the efficacy against *C. albicans* agrees with recent studies highlighting the potential of saponins in antifungal therapy [38].

The efficacy of the ethyl acetate extract against *S. aureus* and its moderate effect against *C. albicans* add to the compound's profile as a selective antimicrobial agent [39,40]. In contrast, the ethanol extracts exhibited lower activity, which may be attributed to the solvent's polarity affecting the solubility of active compounds [41].

Notably, the methanol extracts demonstrated broad-spectrum activity. This agrees with the literature suggesting that methanol may be more effective at extracting antimicrobial compounds than other solvents [42,43]. However, the reduced efficacy against *K. pneumonia* raises questions about the specific interactions between the extract's constituents and bacterial cells [44].

The consistent MIC of 50 µg/mL for extracts soaked in ethanol reflects a lower efficacy, which may be influenced by the solvent's extraction efficiency or the intrinsic resistance of the microorganisms [45]. This highlights the importance of solvent choice in the extraction process, a theme well-established in phytochemical research [46-48].

Our study's suggestion that the extraction method substantially impacts the antimicrobial potency of *Artemisia herba alba* extracts is a significant contribution to the field. Methanol soaking yields the most potent extracts, which could guide future research into optimizing extraction techniques for medicinal plants [49,50].

The variability in efficacy demonstrates the complexity of plant microbe interactions and suggests that extracts can be tailored to specific microbial targets [51,52]. This tailoring could lead to the development of new antimicrobial agents that are more effective against resistant strains, a critical need given the rising concern over antibiotic resistance [53,54].

It is important to note the variability in the literature regarding the antimicrobial properties of *Artemisia herba alba*. While Mehani et al. [26] reported lower antimicrobial activity, our study aligns more closely with the potent bioactivities observed by El-Shatoury et al. [55]. Such discrepancies could be attributed to variations in plant chemotypes, extraction methodologies, or microbial strains used in the assays, as discussed by Atef et al. [31] and Barashkova et al. [56].

The results are significant in the context of increasing antibiotic resistance and the ongoing search for novel antimicrobial compounds. By highlighting the antimicrobial properties of *Artemisia herba alba* extracts, the study adds valuable information to the field of natural product research and antimicrobial therapy. It underscores the importance of traditional medicinal plants as potential sources of new and effective antimicrobial agents. Additionally, the research provides a framework for future studies aiming to isolate and characterize specific bioactive compounds from *Artemisia herba alba*, further advancing our understanding of its therapeutic potential. The study's findings could have implications in developing new, naturally derived antimicrobial drugs, which are crucial in the face of the global challenge of antibiotic resistance.

Conclusions

This study demonstrated that *Artemisia herba alba* possesses a complex array of chemical constituents, particularly within its essential oil. It has shown variable yet significant antibacterial and antifungal activities across different extracts. The results suggest that both the chemical composition and extraction technique are crucial in determining the efficacy of the extracts against various microorganisms. Although this study provides valuable insights into the potential use of A[illegible]misia herba alba as a natural source of antimicrobial agents, the findings are constrained by limitations such as the lack of in[illegible] testing, potential seasonal and geographical variation in plant chemistry, and the need for further toxicological assessment. Future investigations are warranted to explore the full therapeutic potential and safety of these extracts and the mechanisms underlying their bioactivity.

References

1. Boukhennoufa A, Boumediene M, Aicha TT. A study of acute dermal toxicity of Artemisia herba-alba Asso essential oils. Indian Journal of Natural Products and Resources (IJNPR)[Formerly Natural Product Radiance (NPR)]. 2021 Jul 29;12(2):225-9.
2. Dif MM, Fatima zohra D. Antimicrobial Properties and Physical Characteristics of Essential Oil Extracted from Artemisia herba alba Collected in El Bayadh, Algeria. bioRxiv. 2023:2023-04.
3. Amor, G.; Caputo, L.; La Storia, A.; De Feo, V.; Mauriello, G.; Fechtali, T. Chemical Composition and Antimicrobial Activity of Artemisia herba-alba and Origanum majorana Essential Oils from Morocco. Molecules 2019, 24, 4021. https://doi.org/10.3390/molecules24224021
4. Al-Otibi, F.; Alshammry, N.A.; Alharbi, R.I.; Bin-Jumah, M.N.; AlSubaie, M.M. Silver Nanoparticles of Artemisia sieberi Extracts: Chemical Composition and Antimicrobial Activities. Plants 2023, 12, 2093. https://doi.org/10.3390/plants12112093
5. Naz R, Bano A. Phytochemical screening, antioxidants and antimicrobial potential of Lantana camara in different solvents. Asian Pac J Trop Dis. 2013 Dec;3(6):480–6. doi: 10.1016/S2222-1808(13)60104-8. PMCID: PMC4027341.

6. Suriyaprom, S.; Mosoni, P.; Leroy, S.; Kaewkod, T.; Desvaux, M.; Tragoolpua, Y. Antioxidants of Fruit Extracts as Antimicrobial Agents against Pathogenic Bacteria. Antioxidants 2022, 11, 602. https://doi.org/10.3390/antiox11030602
7. Mohammed S, Dekabo A, Hailu T. Phytochemical analysis and anti-microbial activities of Artemisia spp. and rapid isolation methods of artemisinin. AMB Express. 2022 Feb 12;12(1):17. doi: 10.1186/s13568-022-01346-5. PMID: 35150378; PMCID: PMC8840944.
8. Parmanik, A., Das, S., Kar, B. et al. Current Treatment Strategies Against Multidrug-Resistant Bacteria: A Review. Curr Microbiol 79, 388 (2022). https://doi.org/10.1007/s00284-022-03061-7
9. Allemailem, K.S. Aqueous Extract of Artemisia annua Shows In Vitro Antimicrobial Activity and an In Vivo Chemopreventive Effect in a Small-Cell Lung Cancer Model. Plants 2022, 11, 3341. https://doi.org/10.3390/plants11233341
10. Mapoung, S., Semmarath, W., Arjsri, P. et al. Comparative analysis of bioactive-phytochemical characteristics, antioxidants activities, and anti-inflammatory properties of selected black rice germ and bran (Oryza sativa L.) varieties. Eur Food Res Technol 249, 451–464 (2023). https://doi.org/10.1007/s00217-022-04129-1
11. Peng, W., Han, T., Xin, WB. et al. Comparative research of chemical constituents and bioactivities between petroleum ether extracts of the aerial part and the rhizome of Atractylodes macrocephala . Med Chem Res 20, 146–151 (2011). https://doi.org/10.1007/s00044-010-9311-8
12. Tharib, S. M. and El-Migirab, S.I. 1984 Physiochemical investigations on natural products. First Edition. California
13. An, Min; Pratley, J E; Haig, T. Phytotoxicity of Valpiar residues: I. Investigation of aqueous extract. Journal of Chemical Ecology; New York Vol. 23, Iss. 8, (Aug 1997): 1979-1995. DOI:10.1023/B:JOEC.0000006484.57119.84
14. Al-Charchafchi, F.M.R. and Mahnood A. Clor (1997) Germination and seedling development of Atriplex canescense as affected by endogenous chemical inhibitors. Proc. 8th Conf. of Arab Biologists, Amman, Jordan
15. Derwich E, Benziane Z, Boukir, A, Australian J. Basic and Applied Sci., 2009, 3, 3818.
16. Woerdenbag H, Bos R, Salomons C, Hendriks H, Pras, N, Malingre T, Flav. Fragr. J., 1993, 8, 131.
17. Jansen, A.M., Cheffer, J.J.C., Svendsen, A,B. 1987. Antimicrobial activity of essential oils: a 1976-1986 literature review. Aspects of test methods. Planta Med., 40:395-398.
18. Bauer, A.W., Kirby, E., and Sherris, E.M., Turk, M. 1966 Antibiotic by standardized single disk method. Am. J. Clin. Path., 45:493-496.
19. Nawal Ouguirti, Fouad Bahri , Ahmed Bouyahyaoui and Juergen Wanner. Chemical characterization and bioactivities assessment of Artemisia herba alba essential oil grown in South-western Algeria. Nat. Volatiles & Essent. Oils, 2021; 8(2): 27-36. DOI: 10.37929/nveo.844309

20. Houti, H.; Ghanmi, M.; Satrani, B.; Mansouri, F.E.; Cacciola, F.; Sadiki, M.; Boukir, A. Moroccan Endemic Artemisia herba-alba Essential Oil: GC-MS Analysis and Antibacterial and Antifungal Investigation. Separations 2023, 10, 59. https://doi.org/10.3390/separations10010059
21. Brendler T, Chua LS, Abdullah LC, Yam MF. Editorial: Developing medicinal plant extracts commercially: The importance of extraction and fractionation. Front Pharmacol. 2023 Jan 23;14:1140783. doi: 10.3389/fphar.2023.1140783. PMID: 36755954; PMCID: PMC9900108.
22. Osman Adam Osman Adam*, Ragaa Satti Mohmmed Abadi, Saad Mohamed Hussein Ayoub. The Effect of Extraction method and Solvents on yield and Antioxidant Activity of Certain Sudanese Medicinal Plant Extracts. JPHYTO 2019; 8(5): 248-252. doi: 10.31254/phyto.2019.8507.
23. Liu, X., Renzengwangdui, Tang, S. et al. Metabolomic analysis and antibacterial and antioxidant activities of three species of Artemisia plants in Tibet. BMC Plant Biol 23, 208 (2023). https://doi.org/10.1186/s12870-023-04219-6
24. Bisht, D., Kumar, D., Kumar, D. et al. Phytochemistry and pharmacological activity of the genus artemisia. Arch. Pharm. Res. 44, 439–474 (2021). https://doi.org/10.1007/s12272-021-01328-4
25. Ahameethunisa, A.R., Hopper, W. Antibacterial activity of Artemisia nilagirica leaf extracts against clinical and phytopathogenic bacteria. BMC Complement Altern Med 10, 6 (2010). https://doi.org/10.1186/1472-6882-10-6
26. Mehani, M., Segni, L., Terzi, V. et al. Antifungal activity of Artemisia herba-alba on various Fusarium . Phytothérapie (2016). https://doi.org/10.1007/s10298-016-1071-2
27. Boy, FR; Casquete, R.; Martínez, A.; Córdoba, M.d.G.; Ruíz-Moyano, S.; Benito, M.J. Antioxidant, Antihypertensive and Antimicrobial Properties of Phenolic Compounds Obtained from Native Plants by Different Extraction Methods. Int. J. Environ. Res. Public Health 2021, 18, 2475. https://doi.org/10.3390/ijerph18052475
28. Ecevit, K.; Barros, A.A.; Silva, J.M.; Reis, R.L. Preventing Microbial Infections with Natural Phenolic Compounds. Future Pharmacol. 2022, 2, 460-498. https://doi.org/10.3390/futurepharmacol2040030
29. Simonetti, G.; Brasili, E.; Pasqua, G. Antifungal Activity of Phenolic and Polyphenolic Compounds from Different Matrices of Vitis vinifera L. against Human Pathogens. Molecules 2020, 25, 3748. https://doi.org/10.3390/molecules25163748
30. Appell, M., Tu, YS., Compton, D.L. et al. Quantitative structure-activity relationship study for prediction of antifungal properties of phenolic compounds. Struct Chem 31, 1621–1630 (2020). https://doi.org/10.1007/s11224-020-01549-1
31. Atef, N.M., Shanab, S.M., Negm, S.I. et al. Evaluation of antimicrobial activity of some plant extracts against antibiotic susceptible and resistant bacterial strains causing wound infection. Bull Natl Res Cent 43, 144 (2019). https://doi.org/10.1186/s42269-019-0184-9
32. Karnwal, A. In vitro antibacterial activity of Hibiscus rosa sinensis, Chrysanthemum indicum, and Calendula officinalis flower extracts against Gram negative and Gram-positive food poisoning

bacteria. ADV TRADIT MED (ADTM) 22, 607–619 (2022). https://doi.org/10.1007/s13596-021-00562-x

33. Chassagne F, Samarakoon T, Porras G, Lyles JT, Dettweiler M, Marquez L, Salam AM, Shabih S, Farrokhi DR, Quave CL. A Systematic Review of Plants With Antibacterial Activities: A Taxonomic and Phylogenetic Perspective. Front Pharmacol. 2021 Jan 8;11:586548. doi: 10.3389/fphar.2020.586548. PMID: 33488385; PMCID: PMC7821031.
34. Kose, Merve D., and Oguz Bayraktar. "Extraction of Saponins from Soapnut (Sapindus Mukorossi) and Their Antimicrobial Properties." World Journal of Research and Review, vol. 2, no. 5, May. 2016.
35. Rai, S.; Acharya-Siwakoti, E.; Kafle, A.; Devkota, H.P.; Bhattarai, A. Plant-Derived Saponins: A Review of Their Surfactant Properties and Applications. Sci 2021, 3, 44. https://doi.org/10.3390/sci3040044
36. Ajlouni, A.-W.; Hamdan, E.H.; Alshalawi, R.A.E.; Shaik, MR; Khan, M.; Kuniyil, M.; Alwarthan, A.; Ansari, M.A.; Khan, M.; Alkhathlan, H.Z.; et al. Green Synthesis of Silver Nanoparticles Using Aerial Part Extract of the Anthemis pseudocotula Boiss. Plant and Their Biological Activity. Molecules 2023, 28, 246. https://doi.org/10.3390/molecules28010246
37. Srivastava RP, Kumar S, Singh L, Madhukar M, Singh N, Saxena G, Pandey S, Singh A, Devkota HP, Verma PC, Shiva S, Malik S, Rustagi S. Major phenolic compounds, antioxidant, antimicrobial, and cytotoxic activities of Selinum carvifolia (L.) collected from different altitudes in India. Front Nutr. 2023 Jul 13;10:1180225. doi: 10.3389/fnut.2023.1180225. PMID: 37521418; PMCID: PMC10382142.
38. Saqallah FG, Hamed WM, Talib WH, Dianita R, Wahab HA. Antimicrobial activity and molecular docking screening of bioactive components of Antirrhinum majus (snapdragon) aerial parts. Heliyon. 2022 Aug 27;8(8):e10391. doi: 10.1016/j.heliyon.2022.e10391. PMID: 36072262; PMCID: PMC9441312.
39. Anggraeni, Ghea Ayu Putri; Kartosentono, Sugijanto; Sugijanto, Noor Erma Nasution. Antimicrobial Activity Test of the Extract of n-Hexane, Ethyl Acetate, and 96% Ethanol of Caulerpa racemosa Against Escherichia coli, Staphylococcus aureus, and Candida albicans. Journal of Computational and Theoretical Nanoscience, Volume 18, Numbers 1-2, February 2021, pp. 408-412(5). DOI: https://doi.org/10.1166/jctn.2021.9540
40. Okla, MK; Alatar, A.A.; Al-amri, S.S.; Soufan, WH; Ahmad, A.; Abdel-Maksoud, M.A. Antibacterial and Antifungal Activity of the Extracts of Different Parts of Avicennia marina (Forssk.) Vierh. Plants 2021, 10, 252. https://doi.org/10.3390/plants10020252
41. Hanafy, S.M., Abd El-Shafea, Y.M., Saleh, W.D. et al. Chemical profiling, in vitro antimicrobial and antioxidant activities of pomegranate, orange and banana peel-extracts against pathogenic microorganisms. J Genet Eng Biotechnol 19, 80 (2021). https://doi.org/10.1186/s43141-021-00151-0
42. Pandey, A.T., Pandey, I., Kerkar, P. et al. Antimicrobial activity and mycochemical profile of methanol extract from Pleurotus flabellatus. Vegetos 34, 619–629 (2021). https://doi.org/10.1007/s42535-021-00242-w

43. Momeni J, Djialeu Ntchatchoua WP, Fadimatou, Akam MT, Ngassoum MB. Antioxidant activities of some cameroonian plants extracts used in the treatment of intestinal and infectious diseases. Indian J Pharm Sci. 2010 Jan;72(1):140-4. doi: 10.4103/0250-474X.62236. PMID: 20582209; PMCID: PMC2883220.
44. Liang J, Huang X, Ma G. Antimicrobial activities and mechanisms of extract and components of herbs in East Asia. RSC Adv. 2022 Oct 12;12(45):29197-29213. doi: 10.1039/d2ra02389j. PMID: 36320733; PMCID: PMC9554739.
45. Paz Otero, M. Carpena, P. Garcia-Oliveira, J. Echave, A. Soria-Lopez, P. Garcia-Perez, M. Fraga-Corral, Hui Cao, Shaoping Nie, Jianbo Xiao, J. Simal-Gandara & M. A. Prieto (2023) Seaweed polysaccharides: Emerging extraction technologies, chemical modifications and bioactive properties, Critical Reviews in Food Science and Nutrition, 63:13, 1901-1929, DOI: 10.1080/10408398.2021.1969534
46. Indrayanto G. The importance of method validation in herbal drug research. J Pharm Biomed Anal. 2022 May 30;214:114735. doi: 10.1016/j.jpba.2022.114735. Epub 2022 Mar 23. PMID: 35344789.
47. Funari CS, Rinaldo D, Bolzani VS, Verpoorte R. Reaction of the Phytochemistry Community to Green Chemistry: Insights Obtained Since 1990. J Nat Prod. 2023 Feb 24;86(2):440-459. doi: 10.1021/acs.jnatprod.2c00501. Epub 2023 Jan 13. PMID: 36638830.
48. Dey P, Kundu A, Kumar A, Gupta M, Lee BM, Bhakta T, Dash S, Kim HS. Analysis of alkaloids (indole alkaloids, isoquinoline alkaloids, tropane alkaloids). Recent Advances in Natural Products Analysis. 2020:505–67. doi: 10.1016/B978-0-12-816455-6.00015-9. Epub 2020 Mar 20. PMCID: PMC7153348.
49. Mohammed MJ, Anand U, Altemimi AB, Tripathi V, Guo Y, Pratap-Singh A. Phenolic Composition, Antioxidant Capacity and Antibacterial Activity of White Wormwood (Artemisia herba-alba). Plants (Basel). 2021 Jan 16;10(1):164. doi: 10.3390/plants10010164. PMID: 33467047; PMCID: PMC7830657.
50. Hedi Mighri; Hafedh Hajlaoui; Ahmed Akrout; Hanen Najjaa; Mohamed Neffati. Antimicrobial and antioxidant activities of Artemisia herba-alba essential oil cultivated in Tunisian arid zone. Comptes Rendus. Chimie, Volume 13 (2010) no. 3, pp. 380-386. doi : 10.1016/j.crci.2009.09.008. https://comptes-rendus.academie-sciences.fr/chimie/articles/10.1016/j.crci.2009.09.008/
51. Zayda P. Morales Moreira, Melissa Y. Chen, Daniela L. Yanez Ortuno, Cara H. Haney. Engineering plant microbiomes by integrating eco-evolutionary principles into current strategies. Current Opinion in Plant Biology, Volume 71, 2023,102316, https://doi.org/10.1016/j.pbi.2022.102316
52. Diwan D, Rashid MM, Vaishnav A. Current understanding of plant-microbe interaction through the lenses of multi-omics approaches and their benefits in sustainable agriculture. Microbiol Res. 2022 Dec;265:127180. doi: 10.1016/j.micres.2022.127180. Epub 2022 Sep 6. PMID: 36126490.
53. Terreni, M.; Taccani, M.; Pregnolato, M. New Antibiotics for Multidrug-Resistant Bacterial Strains: Latest Research Developments and Future Perspectives. Molecules 2021, 26, 2671. https://doi.org/10.3390/molecules26092671

54. Mancuso, G.; Midiri, A.; Gerace, E.; Biondo, C. Bacterial Antibiotic Resistance: The Most Critical Pathogens. Pathogens 2021, 10, 1310. https://doi.org/10.3390/pathogens10101310
55. El-Shatoury, S.A., Mahmoud, F.M. & El-Kazzaz, W.M. Bioactivities of endophytic actinobacteria inhabiting Artemisia herba-alba emphasizing differences from free-living strains. Folia Microbiol 67, 81–89 (2022). https://doi.org/10.1007/s12223-021-00911-y
56. Barashkova, A.S., Rogozhin, E.A. Isolation of antimicrobial peptides from different plant sources: Does a general extraction method exist?. Plant Methods 16, 143 (2020). https://doi.org/10.1186/s13007-020-00687-1

Supporting information

S1 Fig. *Artemisia herba alba* Asso Composite, the Botanical Garden of Hashemite University, Zarqa, Jordan.

Click here to access/download
Supporting Information
S1 fig.docx
