# Supplementary material for: Seriphidium herba-alba (Asso): A comprehensive study of essential oils, extracts, and their antimicrobial properties
Source: PLoS One. 2024 Apr 25;19(4):e0302329. doi: 10.1371/journal.pone.0302329 (PMC11045107; doi:10.1371/journal.pone.0302329)
Supplement: Attachment — Submitted filename: Rebuttal Letter.docx [file pone.0302329.s007.docx]

Dear Dr. Tripathi,

Thank you for considering our manuscript "*Seriphidium herba-alba (Asso)*: A Comprehensive Study of Essential Oils, Extracts, and Their Antimicrobial Properties" for publication in PLOS ONE. We appreciate the constructive feedback provided by the reviewers and the opportunity to revise our manuscript accordingly.

In response to the reviewers' comments, we have made several revisions to address their concerns and improve the quality of the manuscript. A detailed summary of our responses is provided below:

Reviewer #1:

1. We have corrected the plant name to *Seriphidium herba-alba* (Asso) Y.R. Ling based on the WFO Plant List.
2. The species name has been uniformly formatted as herba-alba throughout the manuscript.
3. All units have been standardized to ensure consistency (e.g., ml to mL).
4. We have carefully revised the manuscript to correct typographical and grammatical errors.
5. Sentences marked for restructuring have been revised for clarity and precision.
6. The term "Methanol extract" has been clarified to refer to "methanolic extract."
7. The results section has been reviewed and amended to ensure appropriate detail for all discussed extracts.
8. All references have been formatted uniformly according to the journal's guidelines. All references have been formatted uniformly according to the journal's guidelines.

Reviewer #2:

1. The abstract has been revised to include specific data for improved informativeness.
2. Accession numbers for pathogens used in the study have been provided in the materials and methods section.
3. Language and syntax have been thoroughly reviewed and corrected.

We believe these revisions have substantially improved the manuscript, aligning it more closely with PLOS ONE's publication criteria. We are confident that the revised version represents a valuable contribution to the field of antimicrobial properties of Seriphidium herba-alba.

Thank you for your consideration, and we look forward to your feedback on the revised manuscript.

Best regards,

Hazem Aqel

Dear Reviewer #1,

I hope this letter finds you well. I sincerely appreciate the time and effort you have dedicated to reviewing my manuscript, "*Seriphidium herba-alba* (Asso): A Comprehensive Study of Essential Oils, Extracts, and Their Antimicrobial Properties" submitted to PLOS ONE Journal. Your insightful comments and suggestions have significantly contributed to improving the manuscript. In response to your valuable feedback, I have made the necessary revisions as outlined below:

1. I have confirmed the accepted plant name as *Seriphidium herba-alba* (Asso) Y.R. Ling, as per the WFO Plant List (wfoplantlist.org).
2. The species name has been corrected to herba-alba throughout the manuscript.
3. Uniform units (ml to mL) have been ensured across the manuscript.
4. Typographical and grammatical errors have been corrected.
5. Sentences have been restructured as suggested.
6. I respectfully disagree with the suggestion to validate the results statistically using a t-test. Given the nature of our study and the available data, statistical validation is deemed unnecessary.
7. Instances of "Methanol extract" have been revised to "methanolic extract" for clarity.
8. The discrepancy regarding a subset of extracts mentioned in line 292 has been addressed by revising the discussion to focus on the appropriate extract.
9. The mention of several extracts in line 295 has been rectified by providing details for each extract mentioned.
10. References have been formatted uniformly and in accordance with the proper journal format.
11. Unfortunately, I cannot provide a clear picture of the habits and habitat of the plant as requested. However, I have included a detailed description in the manuscript.

Once again, I would like to express my gratitude for your thorough review and constructive feedback. I believe these revisions have strengthened the quality and clarity of the manuscript. Please do not hesitate to contact me if further clarification or modifications are needed.

Thank you for your consideration.

Sincerely.

Hazem Aqel

Dear Reviewer #2,

I hope this message finds you well. I would like to express my gratitude for taking the time to review my manuscript titled "*Seriphidium herba-alba* (Asso): A Comprehensive Study of Essential Oils, Extracts, and Their Antimicrobial Properties" submitted to PLOS ONE Journal. Your constructive feedback has been invaluable in refining the quality of the manuscript. Below, I outline the actions taken in response to your insightful comments:

1. I have revised the abstract to include pertinent data, enhancing its impact and aligning it more closely with the format expected for original research articles.
2. Accession numbers for the pathogens mentioned in the materials and methods section have been provided for clarity and completeness.
3. Syntax and typographical errors have been thoroughly checked and rectified to ensure clarity and professionalism throughout the manuscript.
4. While I appreciate the suggestion to add additional references such as <https://doi.org/10.3390/coatings11040484> and <https://doi.org/10.3390/coatings10080761>, I have opted not to include them in this revision as I believe the current references adequately support the content and scope of the manuscript.

Once again, I sincerely appreciate your thoughtful review and constructive feedback. Please let me know if there are any further revisions or clarifications required.

Thank you for your time and consideration.

Regards.

Hazem Aqel
